# Vulnerability to shear stress caused by altered peri-endothelial matrix is a key feature of Moyamoya disease

**DOI:** 10.1038/s41598-021-81282-9

**Published:** 2021-01-15

**Authors:** Muneaki Matsuo, Satomi Nadanaka, Minami Soga, Taku Sugiyama, Shota Serigano, Kenjiro Shimano, Fumio Ichinose, Takuji Nakamura, Toshiyuki Maeda, Kiyohiro Houkin, Takumi Era, Hiroshi Kitagawa

**Affiliations:** 1 Department of Pediatrics, Faculty of Medicine, Saga University, 5-1-1 Nabeshima, Saga, 849-8501 Japan; 2 Laboratory of Biochemistry, Kobe Pharmaceutical University, Kobe, Japan; 3 Department of Cell Modulation, Institute of Molecular Embryology and Genetics, Kumamoto University, Kumamoto, Japan; 4 Department of Neurosurgery, Hokkaido University Graduate School of Medicine, Sapporo, Japan; 5 Department of Mechanical Systems Engineering, Faculty of Engineering, Tokyo City University, Setagaya, Japan

**Keywords:** Glycobiology, Mechanisms of disease, Diseases of the nervous system, Stroke

## Abstract

Moyamoya disease (MMD) is characterized by progressive bilateral stenotic changes in the terminal portion of the internal carotid arteries. Although *RNF213* was identified as a susceptibility gene for MMD, the exact pathogenesis remains unknown. Immunohistochemical analysis of autopsy specimens from a patient with MMD revealed marked accumulation of hyaluronan and chondroitin sulfate (CS) in the thickened intima of occlusive lesions of MMD. Hyaluronan synthase 2 was strongly expressed in endothelial progenitor cells in the thickened intima. Furthermore, MMD lesions showed minimal staining for CS and hyaluronan in the endothelium, in contrast to control endothelium showing positive staining for both. Glycosaminoglycans of endothelial cells derived from MMD and control induced pluripotent stem cells demonstrated a decreased amount of CS, especially sulfated CS, in MMD. A computational fluid dynamics model showed highest wall shear stress values in the terminal portion of the internal carotid artery, which is the predisposing region in MMD. Because the peri-endothelial extracellular matrix plays an important role in protection, cell adhesion and migration, an altered peri-endothelial matrix in MMD may contribute to endothelial vulnerability to wall shear stress. Invading endothelial progenitor cells repairing endothelial injury would produce excessive hyaluronan and CS in the intima, and cause vascular stenosis.

## Introduction

Moyamoya disease (MMD) is characterized by progressive stenotic changes in the terminal portion of the bilateral internal carotid arteries (ICA)^1,2^. These stenotic changes result in the formation of fine collateral vessels (‘moyamoya’ vessels) at the base of the brain. MMD affects both adults and children, and causes both ischemic and hemorrhagic stroke.

Histopathological analyses of stenotic lesions from patients with MMD have shown eccentric fibrocellular thickening of the intima, irregular waving of the internal elastic lamina, and attenuation of the media^3^. Recent genome-wide and locus-specific association studies identified *RNF213* as an important susceptibility gene for MMD^4,5^. However, the exact mechanisms by which abnormalities in *RNF213* lead to MMD remain unknown.

Recent laboratory studies suggest that endothelial progenitor cells (EPCs) may be important in the pathogenesis of MMD^6,7^. A previous study using pathological specimens demonstrated the presence of EPCs within the intima of occlusive arterial lesions in MMD^7^. Intimal thickening, caused by an accumulation of hyaluronan (HA) derived from migrated smooth muscle cells, has been reported to have an important role in the closure of the ductus arteriosus^8,9^. Thus, in the present study we investigated the hypothesis that HA produced by EPCs may be responsible for the intimal thickening in MMD.

During the initial immunohistochemical investigation of the MMD lesion, changes in the endothelial extracellular matrix were demonstrated. Therefore, we hypothesized that an altered vascular endothelial matrix may contribute to the vulnerability of the endothelium to wall shear stress (WSS) in MMD. To confirm this hypothesis, glycosaminoglycans (GAGs) of endothelial cells derived from MMD and control induced pluripotent stem cells (iPSCs) were analyzed. Furthermore, a computational fluid dynamics model was also developed to show the distribution of values for vascular WSS in the predisposing region of MMD.

## Results

### Immunohistochemistry of MMD lesions

**Figure 1:**
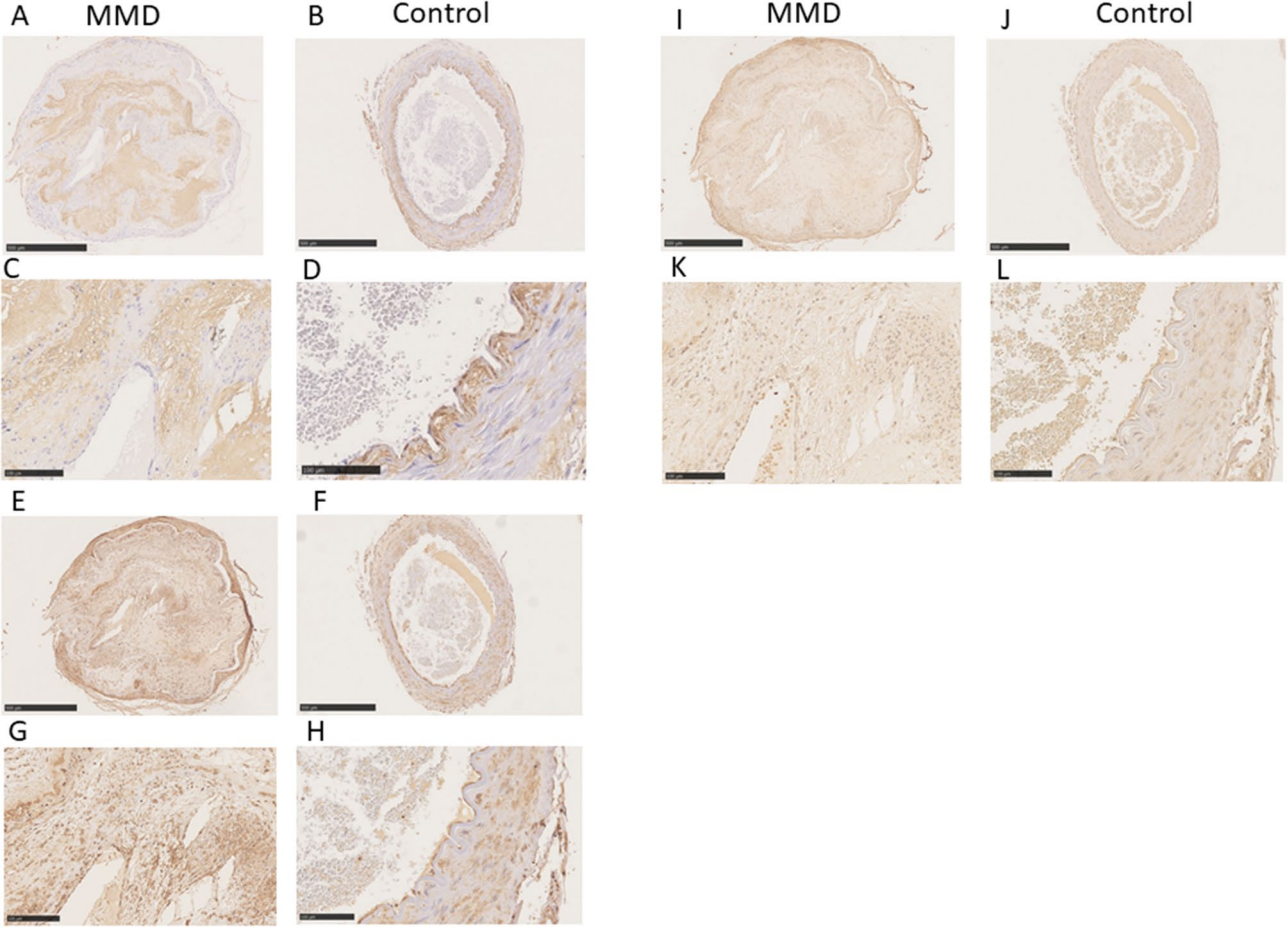
Immunohistochemical staining for HA (panels **A**,**B**,**C**,**D**), HAS2 (panels **E**,**F**,**G**,**H)**, and COX2 (panels **I**,**J**,**K**,**L**). There was strong staining for HA in the thickened intima of the middle cerebral artery from the patient with MMD (**A**,**C**). Specimens from Control 1 (**B**) and Control 2 (**D**) exhibited HA staining on vascular endothelium, which was not stained in the MMD sample (arrows). There was weak staining for HA in the thickened intima of the carotid artery from Control 2 (**D**). There was strong HAS2 staining in the VSMCs in the MMD sample (**E**,**G**) and both Control 1 (**F**) and Control 2 (**H**), as well as in the infiltrating cells within the thickened intima of the MMD specimen (**E**,**G**). VSMCs stained weakly for COX2 in specimens from the patient with MMD (**I**,**K**), Control 1 (**J**), and Control 2 (**L**). In addition, infiltrating cells within the thickened intima of the MMD specimen (**I,K**) stained positive for COX2. Scale bar: 500 μm for (**A**,**B**,**E**,**F**,**I**,**J**); 100 μm for (**C**,**D**,**G**,**H**,**K**,**L**).

**Figure 2:**
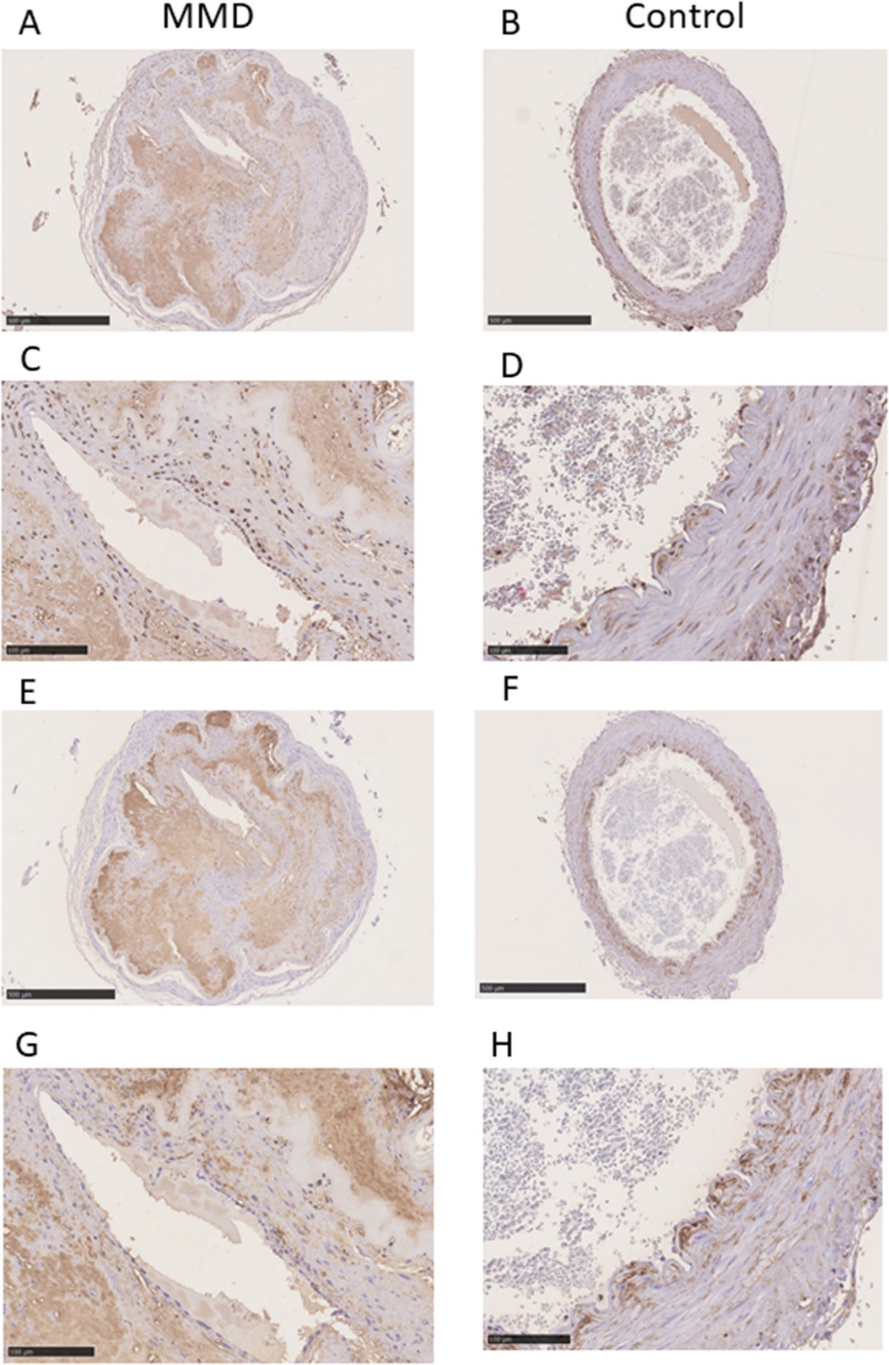
Immunohistochemical staining for CS. In MMD, there was strong staining in the thickened intima, and faint staining in peri-endothelial cells for both CS-C4 (panels **A**,**C**) and CS-C6 (panels **E**,**G**). In contrast, there was stronger staining for both CS-C4 (panels **B**,**D**) and CS- C6 (panels **F**,**H**) in the endothelial cells of controls (arrows). CS-C4, antibodies against chondroitin 4-sulfate (2B6); CS-C6, antibodies against chondroitin 6-sulfate (3B3). Scale bar: 500 μm for (**A**,**B**,**E**,**F**); 100 μm for (**C**,**D**,**G**,**H**).

Staining of samples for HA with HA-binding protein revealed marked accumulation of HA in the thickened intima of a specimen from a patient with MMD, whereas in the control specimens, HA staining was detected in the endothelium and in the outside margins of the internal elastic lamina, with only a small amount of HA detected within the intima. In contrast with the control specimens, there was no staining for HA in the endothelium of the specimen from the patient with MMD (Fig. 1A–D). There was strong staining for HA synthase 2 (HAS2) in the infiltrated cells in the thickened intima, the endothelium, and in vascular smooth muscle cells (VSMCs) in the specimen from the patient with MMD. There was also strong staining for HAS2 in the VSMCs of controls. There was strong HAS2 staining in the endothelium of both control specimens (Fig. 1E–H). There was weak staining for cyclooxygenase 2 (COX2) in the infiltrated cells in the thickened intima and VSMCs of the specimen from the patient with MMD, and comparable COX2 staining in the VSMCs in both control specimens (Fig. 1I–L). There was strong staining for chondroitin sulfate (CS) with C6 sulfation (chondroitin 6-sulfate [CS-C6]) and relatively weak staining for CS with C4 sulfation (chondroitin 4-sulfate [CS-C4]) in the thickened intima and VSMCs of both MMD and control specimens. There was slight, minimal staining for CS-C6 and CS-C4 in the peri-endothelial cells of MMD, whereas in the control specimen there was relative stronger staining for both CS-C6 and CS-C4 in the endothelial cells (Fig. 2).

### Endothelial differentiation of iPSCs derived from MMD and control

**Figure 3:**
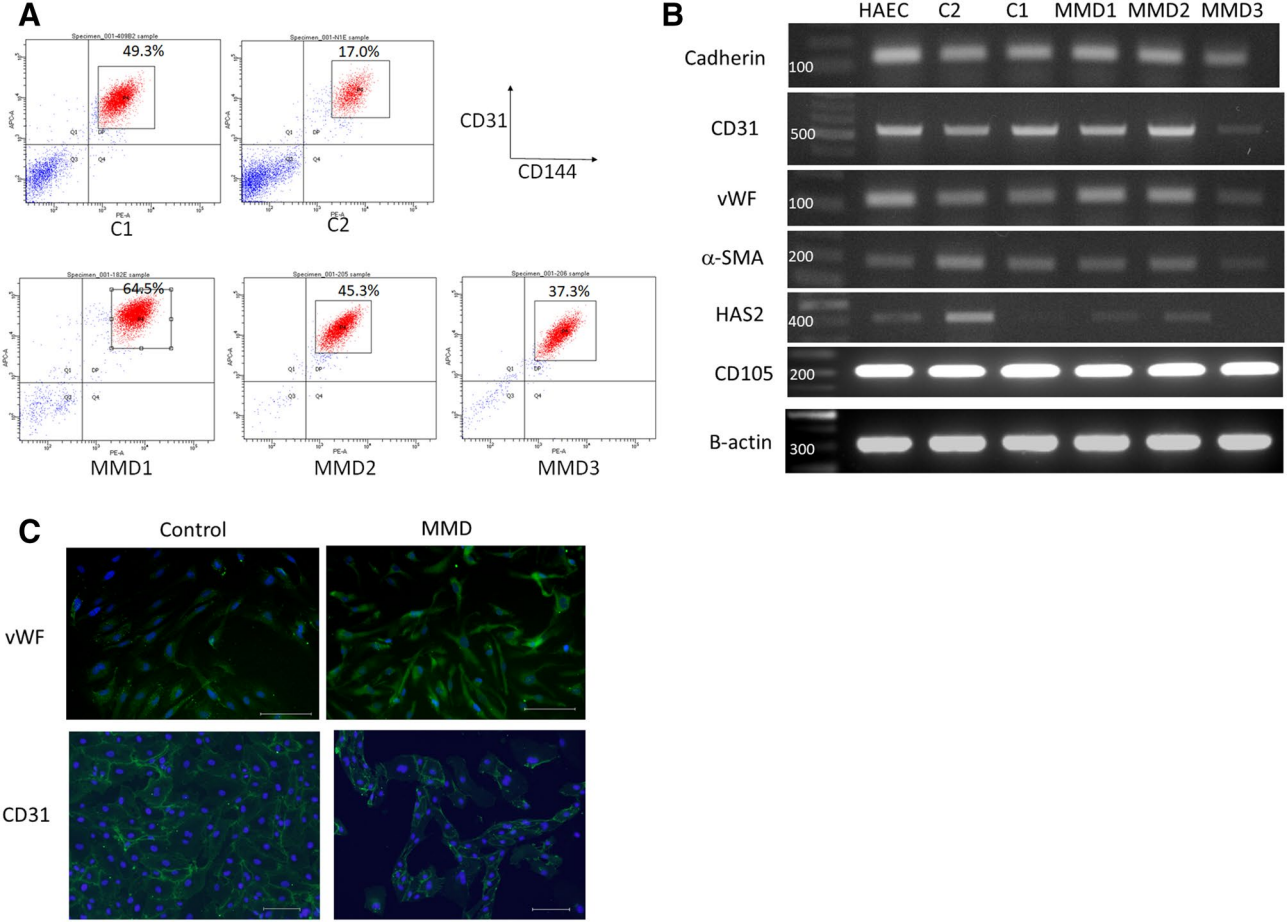
Endothelial differentiation of iPSCs derived from MMD and control. (**A**) FACS analysis of CD144 and CD31 in each differentiated iPSC sample. (**B**) RT-PCR results of endothelial-differentiated iPSCs (iPSECs). All purified cells had the endothelial cell markers, VE-cadherin, vWF, CD31 and CD105, and the smooth muscle cell marker, α-SMA. HAS2 was also expressed all iPSECs except for MMD3. HAEC, human aortic endothelial cell. The original full-length gels were provided as supplementary figures (Figure S3). (**C**) Representative photographs of immunofluorescence for vWF and CD31. Scale bar: 100 μm.

All three patients with MMD had the common *R4810K* mutation of *RNF213*, while control individuals had no *R4810K* mutation in *RNF213* (Figure S1) . After endothelial differentiation, we sorted 17.5%-64.5% of CD31 + CD144 + cells from every iPSC line (Fig. 3A). The purified cells had the endothelial cell markers, VE-cadherin, von Willebrand factor, CD31 and CD105, and the smooth muscle cell marker, α-smooth muscle actin (Fig. 3B, C, S4). We regarded these cells as iPSC-derived endothelial cells (iPSECs).

### Disaccharide analysis of GAGs from iPSECs

**Figure 4:**
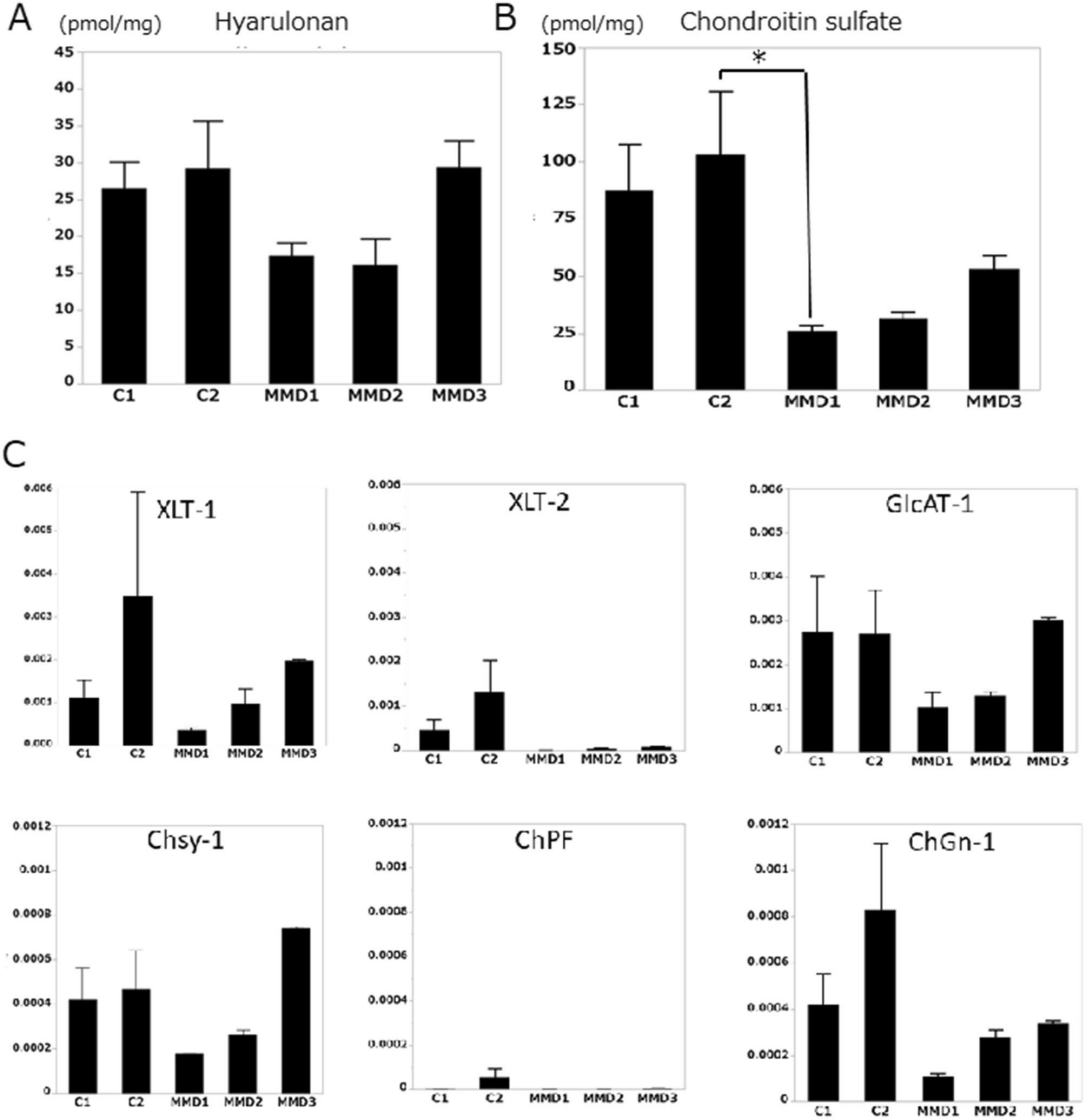
(**A**) The amount of HA in each iPSEC sample. (**B**) The amount of CS produced by each iPSEC sample. MMD1 produced significantly smaller amounts of CS compared with Control 2 (*P < 0.05). (**C**) Expression of enzymes for CS synthesis. Although there was no statistical significance, XLT-2 tended to be downregulated in MMD. XLT, xylose transferase; GlcAT-I, glucuronosyltransferase-I; ChSy-1, chondroitin synthase-1; ChPF, chondroitin polymerizing factor; ChGn, chondroitin β1,4-*N*-acetylgalactosaminyltransferase. Error bars are SEM.

**Figure 5:**
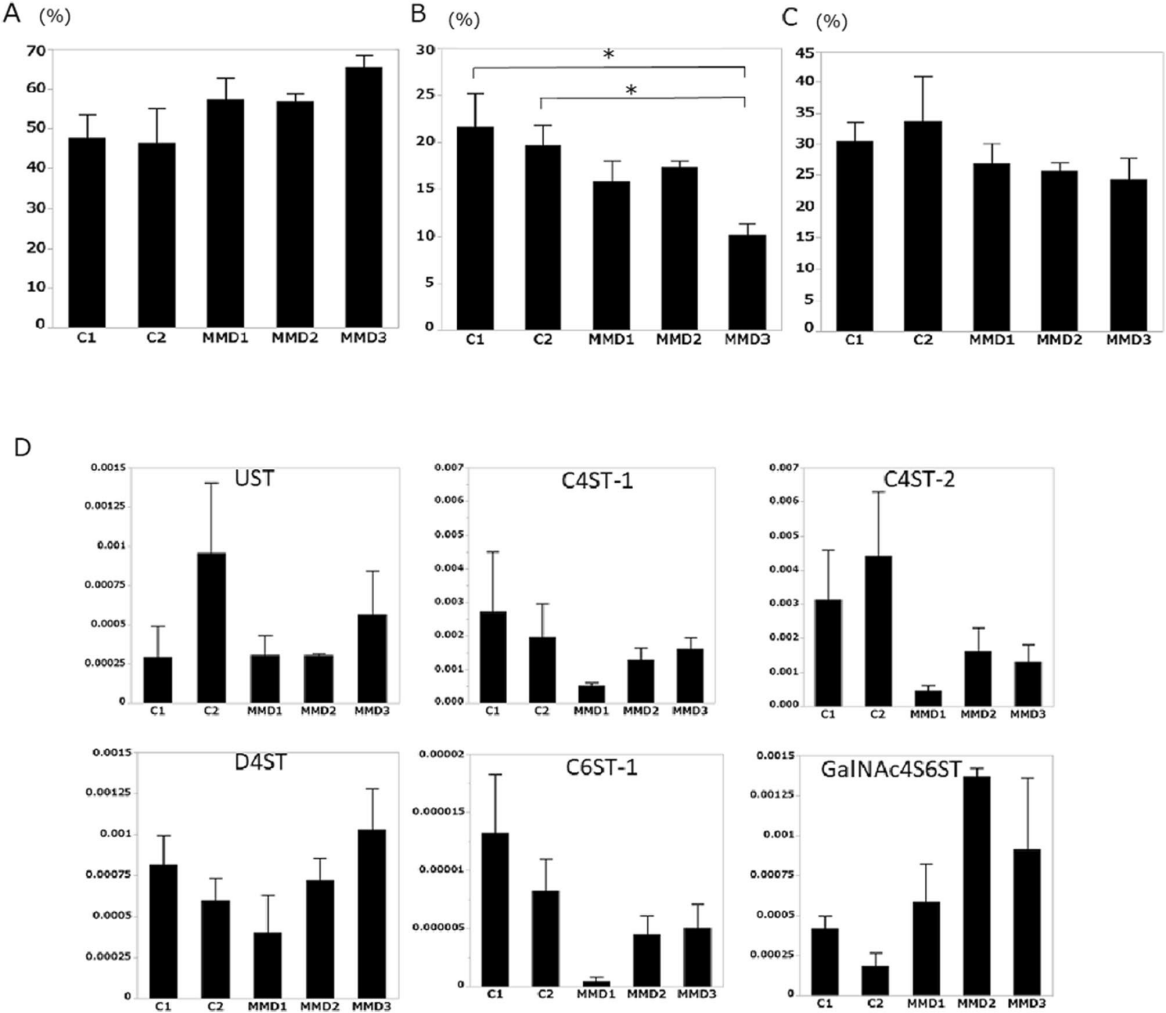
(**A**) Low sulfated CS ratio to total CS for each iPSEC sample. (**B**) CS-C6 ratio to total CS for each iPSEC sample. MMD3 showed a significantly lower ratio of CS-C6, compared with Control 1 or 2 (*P < 0.05). (**C**) CS-C4 ratio to total CS for each iPSEC sample. (**D**) Expression of enzymes for CS sulfation. Although there was no statistical significance, C4ST1, C4ST2 and C6ST-1 tended to be downregulated in MMD. UST, uronyl 2-*O*-sulfotransferase; C4ST, chondroitin 4-*O*-sulfotransferase; D4ST, dermatan 4-*O*-sulfotransferase; C6ST, chondroitin 6-*O*-sulfotransferase; GalNAc4S-6ST, *N*-acetylgalactosamine 4-sulfate 6-*O*-sulfotransferase. Error bars are SEM.

No significant differences were noted in HA between control- and MMD-derived iPSECs (Fig. 4A), whereas the amount of CS was significantly decreased in MMD (Fig. 4B). Furthermore, sulfated CS, especially in the C6 position, was decreased in MMD (Fig. 5A–C). Real time -PCR analysis of enzymes for CS synthesis and CS sulfation showed no significant difference between control and MMD, however expression of xylose transferase-2, chondroitin 4-*O*-sulfotransferase-1, 2 (C4ST-1, 2), and chondroitin 6-*O*-sulfotransferase-1 (C6ST-1) tended to be low in MMD (Figs. 4C, 5D).

**Figure 6:**
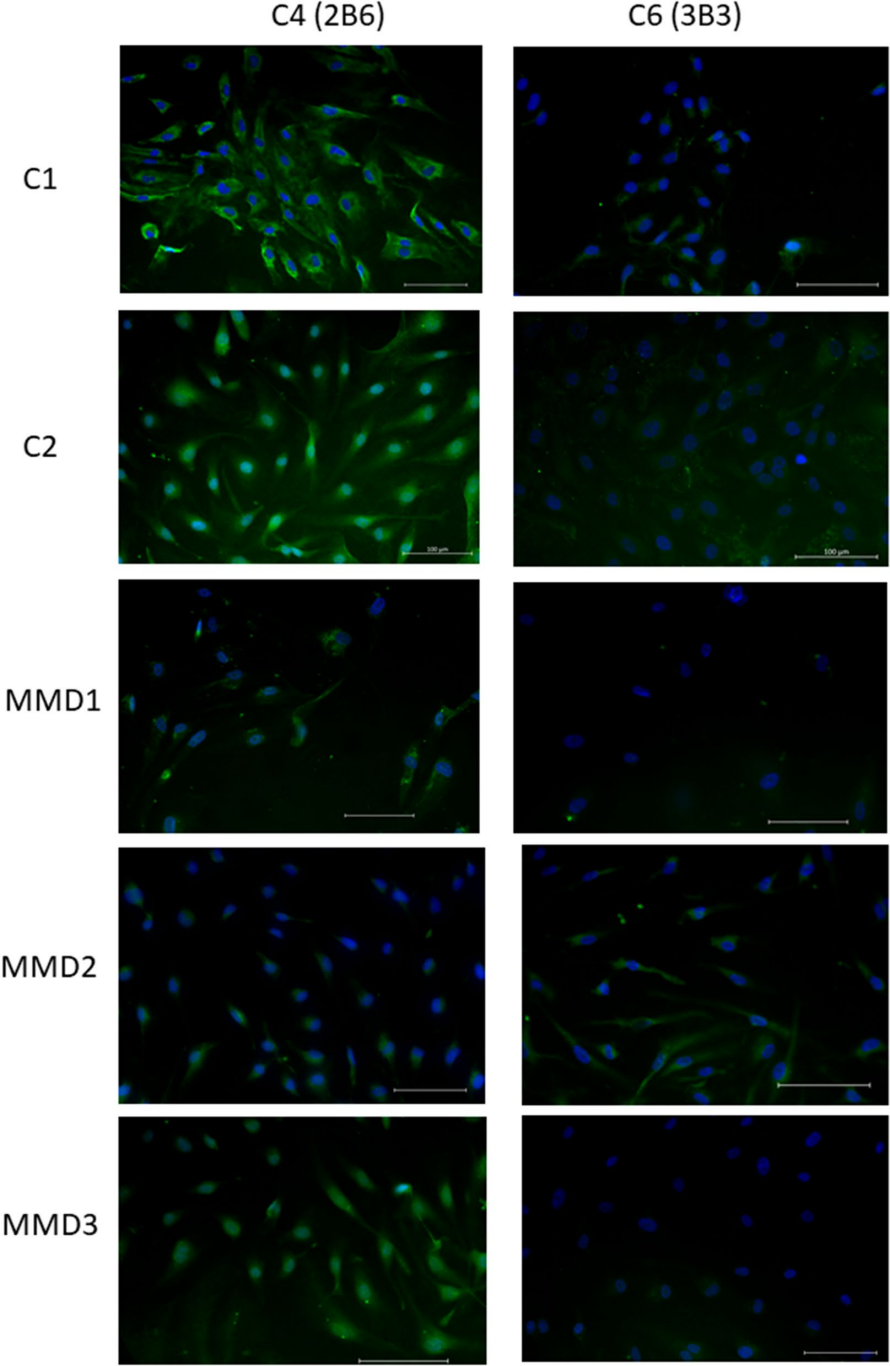
Immunofluorescence for CS-C4 and CS-C6 in endothelial cells derived from MMD and control. MMD1 and MMD2 showed decreased expression of CS-C4 comparing controls. CS-C6 expression was also decreased in MMD1 and MMD3. Scale bar: 100 μm.

Immunofluorescence analysis of iPSECs for CS showed decreased expression of CS-C4 in MMD1 and MMD2 comparing controls. CS-C6 expression was also decreased in MMD1 and MMD3 (Fig. 6).

### Distribution of WSS in a computational fluid dynamics model

**Figure 7:**
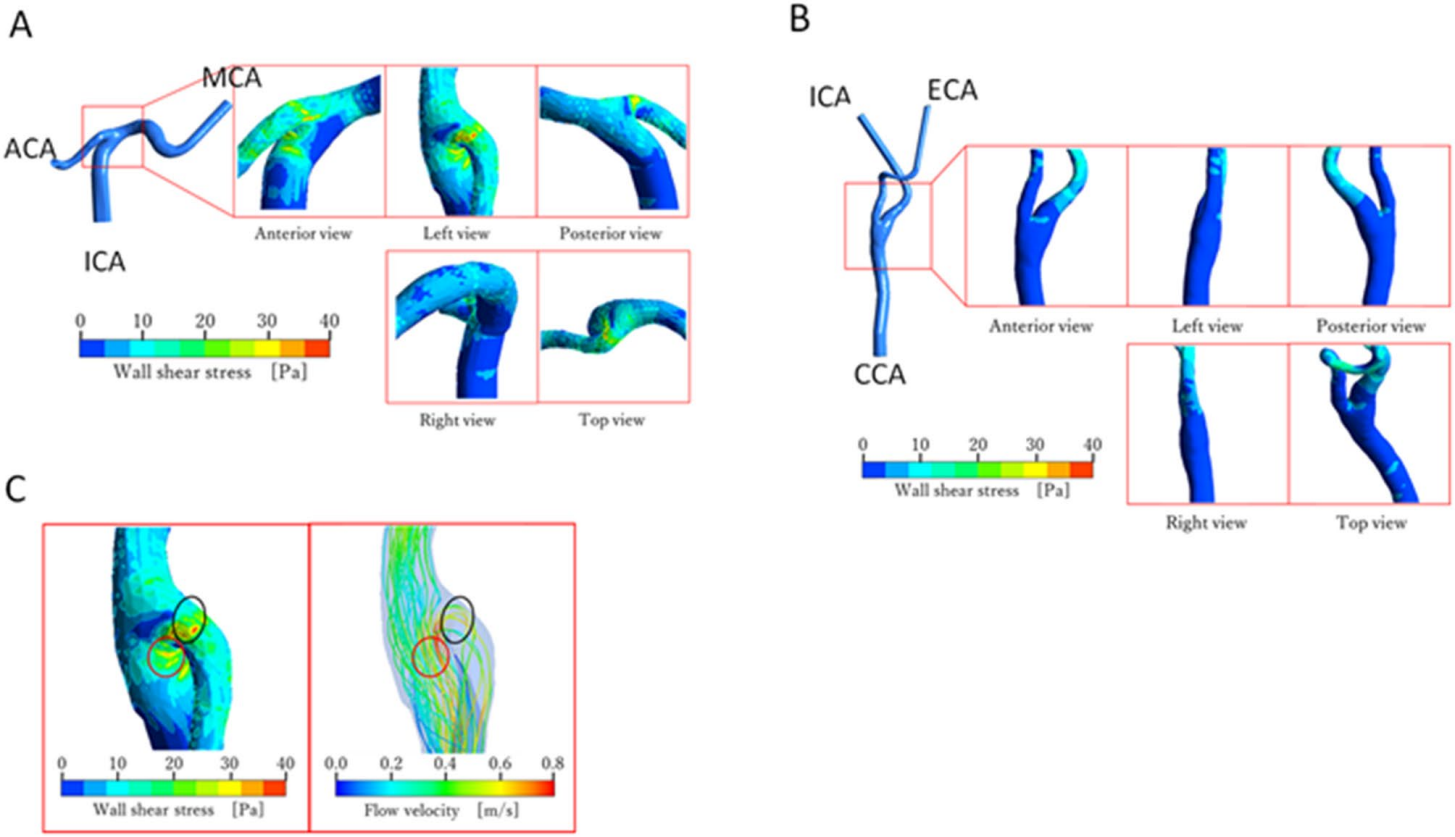
(**A**) WSS distribution around the ICA terminal (model A). ACA, anterior cerebral artery; MCA, middle cerebral artery. (**B**) WSS distribution around the CCA terminal (model B). ECA, external carotid artery. (**C**) Comparison of the WSS distribution with stream lines near the ICA terminal bifurcation (model A).

Distribution of WSS around the ICA terminal (model A) and common carotid artery (CCA) terminal (model B) are shown in Fig. 7A,B, respectively. Note that the same scale of WSS is used in both figures.

It can be clearly seen from the figures that the level of WSS is higher in model A than in model B. The maximum and average magnitudes of WSS around the ICA terminal bifurcation amounted to approximately 38 Pa and 17 Pa, respectively. These values are approximately 3.4 times higher than those around the CCA terminal bifurcation in model B where the maximum and average magnitudes of WSS were up to approximately 11 Pa and 5 Pa, respectively.

Figure 7C compares the WSS distribution (left panel) and stream lines (right panel) in model A. The view angle is the same as in the left view of Fig. 7A. The color of each stream line represents the magnitude of flow velocity. In the right panel of Fig. 7C, blood flowing at high speed is observed near the arterial wall. Such high speed flow is responsible for the high WSS near the ICA terminal bifurcation, because the locations of the high speed flow correspond to the wall surface regions on which high WSS is exerted. This characteristic flow structure was not found near the CCA terminal bifurcation in model B presumably because of the mild branching angle and the bulging shape of the carotid sinus. The observations of the present simulation suggest that the terminal of the ICA, the most frequent site for stenosis in MMD, is subject to high WSS.

## Discussion

The results of the immunohistochemical analysis showed marked accumulation of HA within the thickened intima, accompanied by increased HAS2 and COX2 expression in the infiltrated EPCs, in the occlusive lesion of the patient with MMD. Prostaglandin E_2_ plays an important role in the closure of the ductus arteriosus. Prostaglandin E_2_ dilates vessels by relaxing vascular smooth muscle and forms an intimal cushion by promoting HA synthesis through the induction of HAS2 in the VSMCs migrating into the intima^9^. In a previous study, it was shown that the infiltrating cells in the thickened intima of patients with MMD are bone marrow-derived EPCs, which are involved in vascular repair and remodeling^7^. In the present study, HAS2 expression in VSMCs did not differ significantly between the patient with MMD and the controls, and this HA derived from VSMCs would not be able to enter the intima because of the internal elastic lamina. Thus, in MMD, the HA responsible for intimal thickening is most likely produced by infiltrating EPCs.

HAS2 can be induced by certain proinflammatory cytokines (e.g. interleukin-1 and tumor necrosis factor-α), mechanical stress, and COX2-derived prostaglandins^10,11^. Because the terminal portion of the ICA is subjected to very large hemodynamic stress, as shown in our computational simulation, HAS2 could be strongly induced in this region. Once stenosis occurs by intimal thickening, hemodynamic stress become much greater, and a vicious circle is formed. Furthermore, cerebral infarctions associated with MMD are sometimes triggered by infection (e.g. influenza). Inflammatory cytokines and prostaglandins may also contribute to intimal thickening by inducing HAS2. Interestingly, overexpression of HAS2 has been reported in Down syndrome, which is one of the underling diseases of quasi-MMD^12^.

Immunohistochemical analysis also demonstrated decreased staining of HA and CS in the endothelium of MMD. Especially, both CS-C4 and CS-C6 were decreased in the endothelium of MMD. Quantitative analysis using iPSECs derived from control and MMD patients showed decreased amounts of sulfated CS in MMD, which was confirmed by immunofluorescent staining. Enzyme expression related to CS synthesis and sulfation also tended to be decreased in the iPSECs of patients with MMD.

Decreased expression of C4ST-1 and its relationship to the pathogenesis of Costello syndrome has been reported^13^. Costello syndrome is caused by mutations of *HRAS*, a proto-oncogene, and is one of the underlying diseases of quasi-MMD^14^. Increased *HRAS* signaling causes down-regulation of C4ST-1 expression, and results in cell proliferation and defects in elastic fiber formation in Costello syndrome. Interestingly, other diseases which increase *RAS* signaling, such as Noonan syndrome and neurofibromatosis type 1, are also sometimes complicated with quasi-MMD^15^. Thus, decreased C4ST-1 and hyposulfation could be involved in the pathogenesis of MMD.

The endothelium synthesizes and secretes GAGs, such as heparan sulfate, CS, and HA^16^. Some of these GAGs are deposited into the peri-endothelial extracellular matrix, where they are involved in various functions, such as cell proliferation, adhesion, migration, protection, and signal transduction^16,17^. Furthermore, changes in the sulfation of CS profoundly influence its function, such as inflammation and angiogenesis^18,19^. Downregulation of extracellular matrix receptor-related genes was demonstrated by DNA microarray analysis using iPSECs of MMD^20^. These changes in the extracellular matrix could cause the arterial endothelium to become vulnerable to WSS, and cause invasion of EPCs into the intima to repair the damaged endothelium. Our computational simulation has indicated that the target region for MMD matched the location of high WSS.

**Figure 8:**
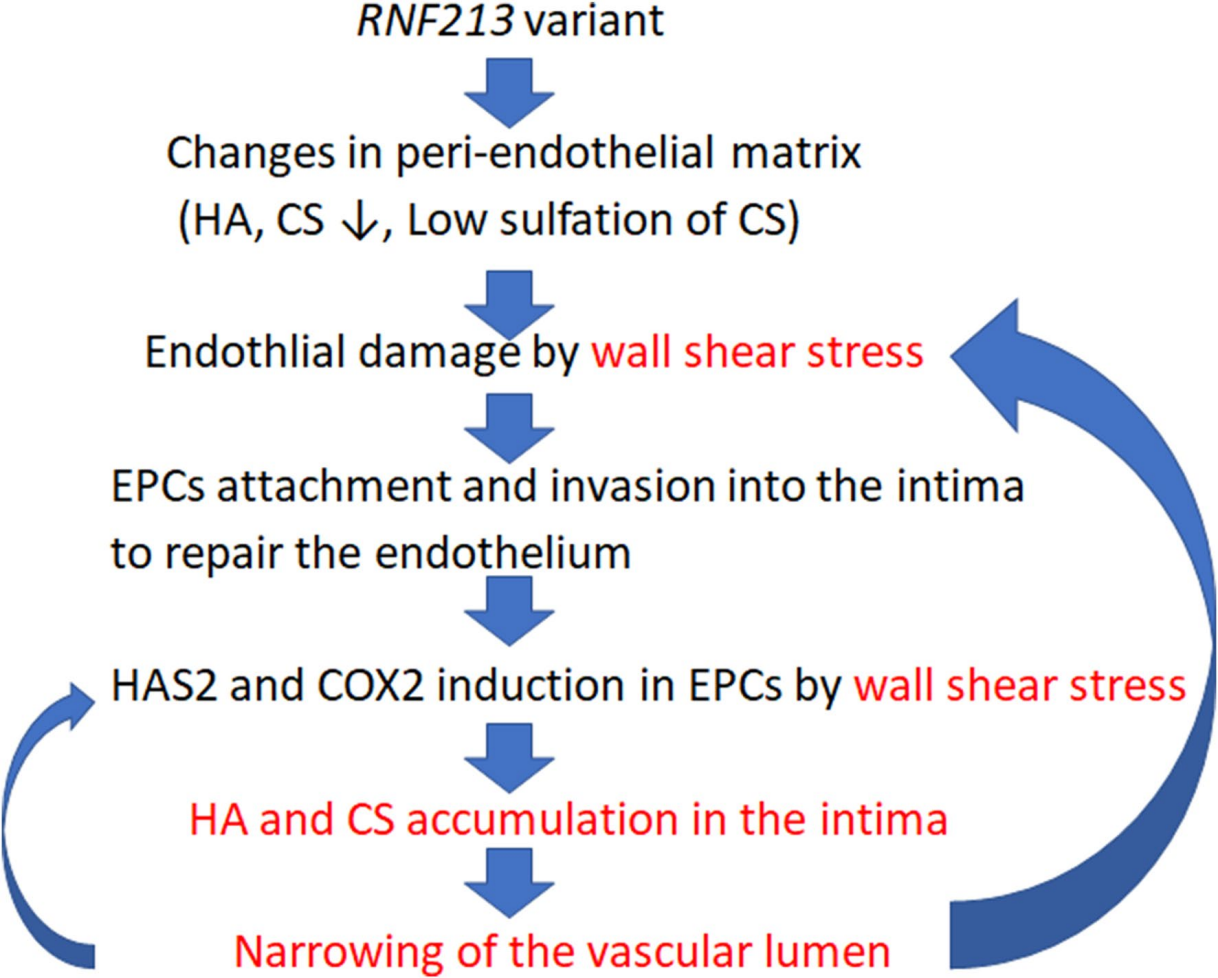
Proposed pathogenesis of vascular occlusion in MMD. Under normal conditions, the intact peri-endothelial matrix protects against vascular shear stress by relaxing VSMCs through the enhanced production of nitric oxide. In MMD, decreased production of CS leads to changes in the peri-endothelial matrix, including decreased HA, which makes the endothelium susceptible to vascular shear stress. EPCs infiltrate through the damaged endothelium, with HAS2 and COX2 subsequently induced in EPCs by shear stress or cytokines. The accumulation of HA in the intima, produced by the infiltrated EPCs, results in eccentric thickening of the intima and narrowing of the vascular lumen, which further increases shear stress.

Although it is unknown how *RNF213* contributes to the pathogenesis of MMD, *RNF213* is possible to interact with sulfation of CS according to the computational network analysis GeneMANIA (https://genemania.org/, Fig. S5). In this study, we have shown decreased sulfation of CS in iPSECs derived from three genetically independent MMD patients with variant *RNF213*. Thus, in MMD, variant *RNF213* causes changes in CS, and the endothelium becomes susceptible to shear stress, making it easier for EPCs to invade the vascular intima, where they would produce HA, resulting in intimal thickening and vascular stenosis. According to the results using a stenotic carotid artery model, WSS would be further increased in the stenotic lesion^21^. Because shear stress induces HAS2 expression, the EPCs that have invaded the intima may be induced to produce even more HA, resulting in a vicious cycle (Fig. 8).

In conclusion, the altered peri-endothelial matrix in patients with MMD may contribute to injury of the vascular endothelium and invasion of EPCs into the intima. HA produced by the infiltrated EPCs cause intimal thickening and vascular obstruction. Further studies would be needed to confirm our hypothesis.

## Methods

### Patients

A 19-year-old female with MMD died as a result of cerebral hemorrhage. An autopsy was performed and specimens of the supraclinoid ICA were obtained. As controls, autopsy specimens were obtained from a 73-year old male with subarachnoid hemorrhage (Control 1) and an 18-year-old male with malignant lymphoma (Control 2). Informed consents from legally authorized representatives/next of kin were obtained for all autopsy samples.

### Immunohistochemistry

To identify HA, specimens were treated with proteinase K (Dako), endogenous peroxidase was blocked, and sections were stained using a biotinylated HA-binding protein (1:50 dilution; Hokudo) at room temperature for 1 h. Sections were then treated with avitin–biotin peroxidase complex solution (Nichirei Biosciences Inc.) and 3,3′-diaminobenzidine (Dako) according to the manufacturer’s instructions.

To detect CS, specimens were treated with 1.25 U/ml of chondroitinase ABC (Sigma-Aldrich), and sections were incubated with primary antibodies against CS-C4 (2B6) and CS-C6 (3B3) (1:20 dilution; Cosmo Bio. Ltd). To detect HAS2 and COX2, sections were incubated with primary antibodies against HAS2 (1:100 dilution; Abgent Biotech) and COX2 (1:100 dilution; Cayman Chemical Co.).

After microwave antigen retrieval and blockade of endogenous peroxidase, immunostaining was performed with the appropriate primary antibody at room temperature for 2 h. Sections were then treated with EnVision (Dako), according to the manufacturer’s instructions, as well as 3,3′-diaminobenzidine. Sections were counterstained with hematoxylin.

### iPSCs

MMD-specific iPSCs were derived from three unrelated patients with MMD. The gender and age of the patients with MMD were as follows: MMD 1 (A182), 6-year-old girl; MMD2 (A205), 3-year-old girl; and MMD3 (A206), 37-year-old woman. Their diagnoses were based on criteria from the Japanese Research Committee on MMD (Ministry of Health, Labour and Welfare, Japan). The disease staging for all three patients were stage 2. Informed consent was obtained from a parent and /or legal guardian as minors (6 year and 3 year). For control iPSCs, the following two cell lines were used: C1 (409B2), purchased from Riken BRC Cell Bank; and C2 (N1), derived from a 48-year-old man.

### iPSC generation

Mononuclear cells were isolated by gradient centrifugation with Ficoll-Paque, and then activated and expanded in KBM502 medium (Kohjin Bio Co.) on anti-CD3 antibody-coated dishes (eBioScience). iPSCs were generated from activated mononuclear cells as described previously^22^. In brief, 5 × 10^5^ mononuclear cells were infected with Sendai virus carrying *OCT3/4*, *SOX2*, *KLF4*, and *c-MYC* at a multiplicity of infection of 10. Sendai virus was prepared as described previously^23^. After 2 days of culture, the infected cells were seeded at 2 × 10^4^ cells per 10 cm dish on mitomycin C-treated mouse embryonic fibroblasts. On the next day, the medium was replaced with iPSC medium. From 15 to 17 days after infection, the colonies were selected and expanded on mouse embryonic fibroblasts with iPSC medium.

### Endothelial differentiation of iPSCs

Endothelial differentiation was performed as described previously with some modifications^24^. The iPSCs were cultured in mTeSR1 medium (Stemcell Technologies) on dishes coated with iMatrix511 (Nippi; (0.5 µg/cm^2^). After the colony size was grown up to 750–1000 μm in diameter, the medium was changed to Essential8 medium (Thermo Fisher Scientific) containing 4 μM of CHIR99021 (Wako), 80 ng/mL of BMP4 (HumanZyme), and 80 ng/mL of VEGF (Thermo Fisher Scientific). Then 48 to 51 h later, cells were detached using TrypLE Express (Thermo Fisher Scientific) and seeded onto dishes coated with LM411-E8 (Nippi; 0.4 μg/cm^2^) with a density of 2500 ~ 10,000/cm^2^ in Stempro-34 SFM (Thermo Fisher Scientific) containing 80 ng/ml of VEGF. Four to six days later, cells were detached using TrypLE Express and sorted using Alexa Fluor 647 mouse anti-human CD31 antibody (1 μg/1 × 10^6^ cells; BD Biosciences) and PE mouse anti-human CD144 antibody (0.5 μg/1 × 10^6^ cells; BD Biosciences) with a FACSAria II flow cytometer (BD Biosciences).

### RT-PCR analysis

RT-PCR analysis was performed as described previously^20^. In brief, total RNA was purified with Isogen (Nippon Gene Co.) and transcribed to DNA with Superscript III (Invitrogen) and random primers (Invitrogen). RT-PCR was conducted using QuickTaq (Toyobo), according to the manufacturer’s instructions. The sequences of primers and amplification conditions for the detection of pluripotent markers were designed as described previously. The primers used for OCT3/4, SOX2, KLF4, and c-MYC were designed to detect the expression of endogenous genes but not of transgenes.

To detect the Sendai virus genome, nested RT-PCR was performed. The primers used for RT- PCR to analyze the results of endothelial differentiation are shown in supplementary Table 1.

### DNA isolation and Sanger sequencing

DNA isolation and Sanger sequences were performed as described previously^20^. In brief, cells were treated with Lysis buffer (10 mmol/L Tris–HCL pH 7.5, 10 mmol/L EDTA, 10 mmol/L NaCl, 1 mg/mL proteinase K, 0.5% SDS). Genomic DNA was precipitated with ice-cold 75 mM NaCl in ethanol and suspended with 50 μL of TE buffer (10 mmol/L Tris–HCl, 1 mmol/L EDTA, pH 8.0). Mutation of *RNF213* (*p*.*R4810K*: *rs112735431*, *G* > *A*) was analyzed by direct sequencing. The sequence reactions were performed using a Big Dye Terminator cycle sequencing kit (Life Technologies) and analyzed using an ABI PRISM 310 Genetic Analyzer (Applied Biosystems). The sequences of primers for detecting *R4810K* in *RNF213* were as follows: forward, 5′-AAAGTTCCTGCCTGAGATTTTG-3′, reverse, 5′-AAATGCGGGACAGTCCTGGT-3’.

### Disaccharide analysis of GAGs from endothelial cells derived from MMD and control iPSCs

Endothelial cells (3 × 10^4^) derived from MMD and control iPSCs were seeded onto 35 mm dishes and cultured for 24 h, and cells were harvested with a scraper into a tube. After centrifugation, supernatant medium was carefully removed, and the residual cells were weighed.

GAGs were isolated and purified from the cells as described previously^25^. Briefly, cells were homogenized and extracted with acetone three times, and air-dried thoroughly. The dried materials were digested with heat-activated actinase E (10% by weight of dried materials) in 0.1 M borate-sodium, pH 8.0, containing 10 mM CaCl_2_ at 55 °C for 48 h. The samples were adjusted to 5% trichloroacetic acid and centrifuged. The resultant supernatants were extracted with diethyl ether three times to remove trichloroacetic acid, and then neutralized using 20% NH_4_HCO_3_. The aqueous phase containing 5% sodium acetate was adjusted to 80% ethanol and left overnight at − 30 °C. The resultant precipitate was dissolved in H_2_O, and subjected to gel filtration on a PD-10 column (GE Healthcare) using H_2_O as an eluent. The flow-through fractions were collected and evaporated to dryness. Purified GAGs were digested with chondroitinase ABC from *Proteus vulgaris* (EC 4.2.2.4) (10 mIU) or with hyaluronidase SD from *Streptococcus dysgalactiae* (EC 4.2.2) (5 mIU) at 37 °C for 4 h. The digests were derivatized with a fluorophore 2-aminobenzamide and then analyzed by high performance liquid chromatography as reported previously^25^.

### Real-time PCR analysis

Quantitative real-time PCR was conducted using FastStart Essential DNA Green Master and a LightCycler 96 (Roche Applied Science) according to the manufacturer’s protocols. The housekeeping gene *GAPDH* was used as an internal control for quantification. The primers used for RT-PCR are shown in supplementary Table 2.

### Immunofluorescent analysis of endothelial cells derived from MMD and control iPSCs

Cells were fixed with 4% paraformaldehyde (Wako). To detect CS, specimens were treated with 1.25 U/ml of chondroitinase ABC (Sigma-Aldrich), and sections were incubated with primary antibodies against CS-C4 (2B6) and CS-C6 (3B3) (1:20 dilution; Cosmo Bio. Ltd). Then, Alexa 488 anti-mouse IgG (Abcam) was used for the secondary antibody.

### Computational fluid dynamics model

Two 3-dimensional models of arteries were made from computed tomography data of a 66-year-old female patient, using a slice thickness of 0.5 mm so that blood flow in the model lumens could be simulated by computational fluid dynamics. As shown in supplemental Figure S2, the two models (model A and model B) represented the terminal bifurcation of the left ICA and that of the left CCA, respectively. The software used for model construction was OsiriX. The mother and daughter arteries in the models were extended by adding straight tubes for the sake of computational fluid dynamics. Computational cells for fluid dynamics were generated in each model by Pointwise V18.2 (Pointwise, Inc.). The number of computational cells was 1891335 in model A and 1858884 in model B. The smallest cell size near the bifurcation was 0.1 mm in model A and 0.2 mm in model B, while cell sizes ranged from 0.3 to 0.4 mm in the regions other than the bifurcations. Although most cells were tetrahedral, four layers of prism cells were arranged over the wall surfaces in both models. Each prism layer had a height of 0.05 mm.

The incompressible Navier–Stokes equations were solved at each computational cell by the ANSYS Fluent Ver.19.0 software. Coupling of the velocity and pressure fields was performed by the SIMPLE algorithm^26^. Blood was treated as a Newtonian fluid with a viscosity of 0.004 Pa s and a density of 1050 kg/m^3^. Steady-state calculation was carried out with the arterial walls assumed to be rigid. Although actual blood flow in the vasculature is pulsatile, steady-state calculation was considered to be sufficient for comparison of the magnitudes of WSS near the terminals of the ICA and CCA. Calculation was terminated after the dimensionless residual of each governing equation had reduced to 10^−6^.

At the proximal opening of model B, the Poiseuille (parabolic) velocity profile with a maximum velocity of 60 cm/s was imposed as the inflow boundary condition. This condition corresponded to an inlet WSS of 1.27 Pa. As the outflow boundary condition, flow rates at the two distal openings were specified with the assumption that flow division at the terminal bifurcation of the CCA followed Murray’s law^27^. In model A, the inflow rate at the ICA was the same as at the terminal of the ICA in model B, because blood passing through the ICA in model B was considered to reach the ICA in model A. Flow division at the bifurcation in model A was also assumed to follow Murray’s law.

### Statistical analysis

Every result is presented as the mean ± SEM. The Wilcoxon test was used to analyze the difference between the means in each group. Statistical significance was accepted at the 95% confidential level (*P* < 0.05). All experiments were repeated at least three times, and representative data are shown.

### Study approval

All patients and control individuals were recruited with written informed consent which was approved by the institutional review board of Saga University Hospital (approval numbers: 22-39). All experimental procedures using human samples were approved by the following ethics committees: Ethics Committee of Saga University Hospital; Ethics Committee for Epidemiological and General Research at the Faculty of Life Science, Kumamoto University; Ethics Committee for Human Genome and Gene Analysis Research at the Faculty of Life Sciences, Kumamoto University; and Ethics Committee for Clinical Research and Advanced Medical Technology, Kumamoto University (approval numbers: 012-0317; 318; 153; and 1018, respectively). All methods were performed in accordance with the relevant guidelines and regulations.

## Supplementary Information

Supplementary Information.

## Data Availability

All data generated or analysed during this study are included in this article and its supplementary information files.
