# Supplementary material for: Vulnerability to shear stress caused by altered peri-endothelial matrix is a key feature of Moyamoya disease
Source: Sci Rep. 2021 Jan 15;11:1552. doi: 10.1038/s41598-021-81282-9 (PMC7810726; doi:10.1038/s41598-021-81282-9)
Supplement: Supplementary file 1 — Supplementary Information. [file 41598_2021_81282_MOESM1_ESM.pdf]

Vulnerability to shear stress caused by altered peri-endothelial matrix is a key feature of Moyamoya disease

Muneaki Matsuo[1*], Satomi Nadanaka[2], Minami Soga[3], Taku Sugiyama[4], Shota Serigano[5], Kenjiro Shimano[5], Fumio Ichinose[1], Takuji Nakamura[1], Toshiyuki Maeda[1], Kiyohiro Houkin[4], Takumi Era[3], Hiroshi Kitagawa[2]

1. Department of Pediatrics, Saga University, Faculty of Medicine, Saga, Japan
2. Laboratory of Biochemistry, Kobe Pharmaceutical University, Kobe, Japan
3. Department of Cell Modulation, Institute of Molecular Embryology and Genetics, Kumamoto University, Kumamoto, Japan
4. Department of Neurosurgery, Hokkaido University Graduate School of Medicine, Sapporo, Japan
5. Department of Mechanical Systems Engineering, Faculty of Engineering, Tokyo City University, Japan

*Correspondence to:

Muneaki Matsuo, MD.

5-1-1 Nabeshima, Saga, 849-8501, Japan

matsuo@cc.saga-u.ac.jp ORCID; 0000-0002-8667-836X

Supplementary Table 1

Primers used for RT-PCR

Gene Name	5'-primer	3'-primer
VE-Cadherin	5'-TGGATGAGAATGACAATGCC-3'	5'-TTGAACTTCACGTTTCGTGG-3'
CD31	5'-AGGTGTTGGTGGAAGGAGTG-3'	5'-CGTGTAGTTGCCACTGTGCT-3'
vWF	5'-AACACCTTTGATGGGAGCAT-3'	5'-TCACTCTCTTGCCATTCTGGA-3'
a-SMA	5'-AGAACATGGCATCATCACCA-3'	5'-TACATGGCTGGGACATGGAA-3'
HAS-2	5'-GTCTCAAATTCATCTGATCTC-3'	5'-ACATTTCCTTAAGTAGTCTGG-3'
CD105	5'- CCACTAGCCAGGTCTCGAAG -3'	5'- GATGCAGGAAGACACTGCTG -3'
Oct-4	5'-GACAGGGGGAGGGGAGGAGCTAGG-3'	5'-CTTCCCTCCAACCAGTTGCCCCAAAC-3'
β-actin	5'- TCCTGTGGCATCCACGAAACT -3'	5'- GAAGCATTTGCGGTGGACGAT -3'

Supplementary Table 2

Primers used for real-time PCR

Gene Name	5’-primer	3’-primer
hXylT1	5’-AAGAAGCCACCGAGTAGAC-3’	5’-TGGACACATCCTTCCCGT-3’
hXylT2	5’-CGCTACAAGCTGGCGAT-3’	5’-ACTGTCTGTGTCCTTGGAAC-3’
hGlcAT-I	5’-CCTGCCTACTATCTATGTTGTTAC-3’	5’-ACCACCAGGTGTGTGAA-3’
hChSy1	5’-GGTTTGGCATCACGTGTAT-3’	5’-ACTCCTACTTCCTGGCTC-3’
hChPF	5’-GACCCTCATTTCCGAAGTG-3’	5’-GCCAGATGGCTGGTATT-3’
hChGn-1	5’-AGAAGAAATAAATGAAGTCAAAGGAATAC-3’	5’-GAAGTAGTAGTCCACATCACAG-3’
hUST	5’-ACCATGGACCACCTCCTAGTAA-3’	5’-CACACTTGCCTACCCTGTTGTA-3’
hC4ST-1	5’-AAACGCCAGCGGAAGAA-3’	5’-GGGATGGCAGAGTGAGTAGA-3’
hC4ST-2	5’-GAGGGAAAGTTCTTTGTTTAAGTG-3’	5’-CGGCCTTAACAGCCATAAT-3’
hC6ST-1	5’-CAGGGGAGGAAGAGGAAGAG-3’	5’-CCCTGCTGGTTGAAGAACTC -3’
hD4ST	5’-ACTCAGAGGACAGGGCTA-3’	5’-ACCAAGTGGACTGCATAATC-3’
hGalNAc4S-6ST	5’-TCGTGTGGACAGTAAGCAGAT-3’	5’-TGTAAGAAGCCATTACCAAGGTC-3’
hGAPDH	5’-ATGGGTGTGAACCATGAGAAGTA-3’	5’-GGCAGTGATGGCATGGAC -3’

Figure S1. DNA analyses for RNF213 RNF213 SNP : G>A ss179362673

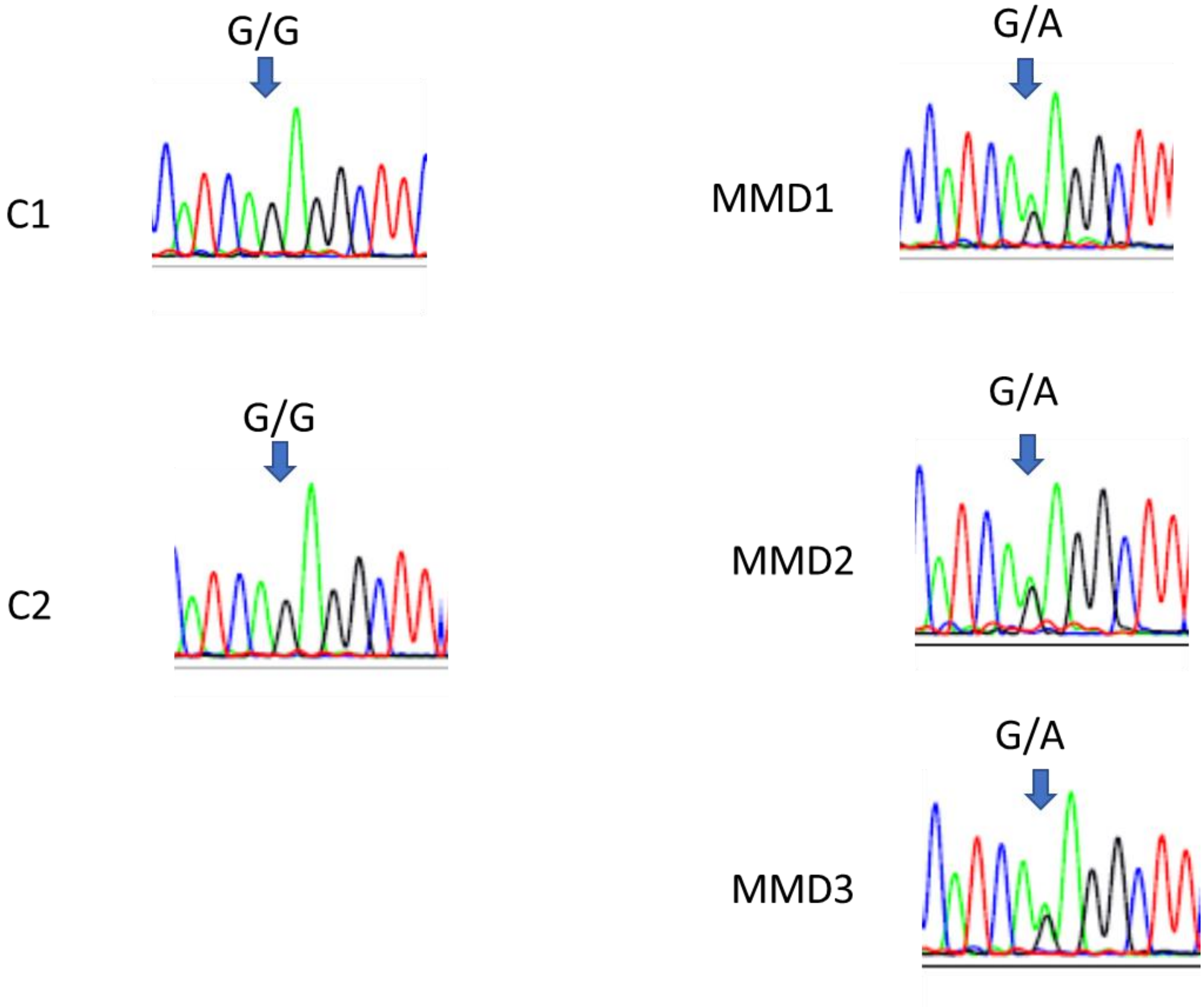

Figure S2. Three-dimensional models of arterial branches.

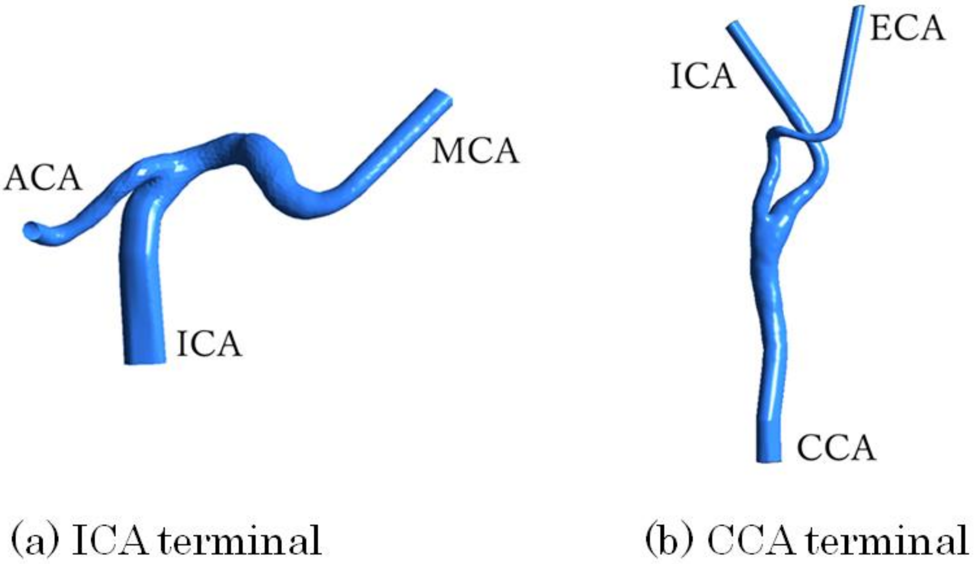

Figure S3. Biosynthesis of chondroitin sulfate.

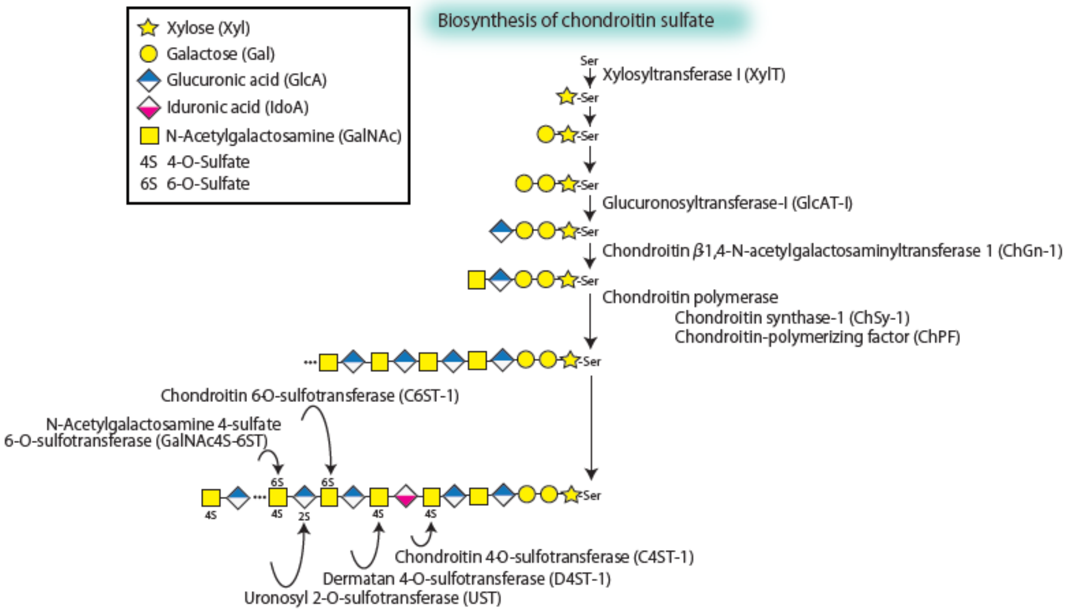

Figure S4. RT-PCR results of endothelial-differentiated iPSCs

A. Cadherin

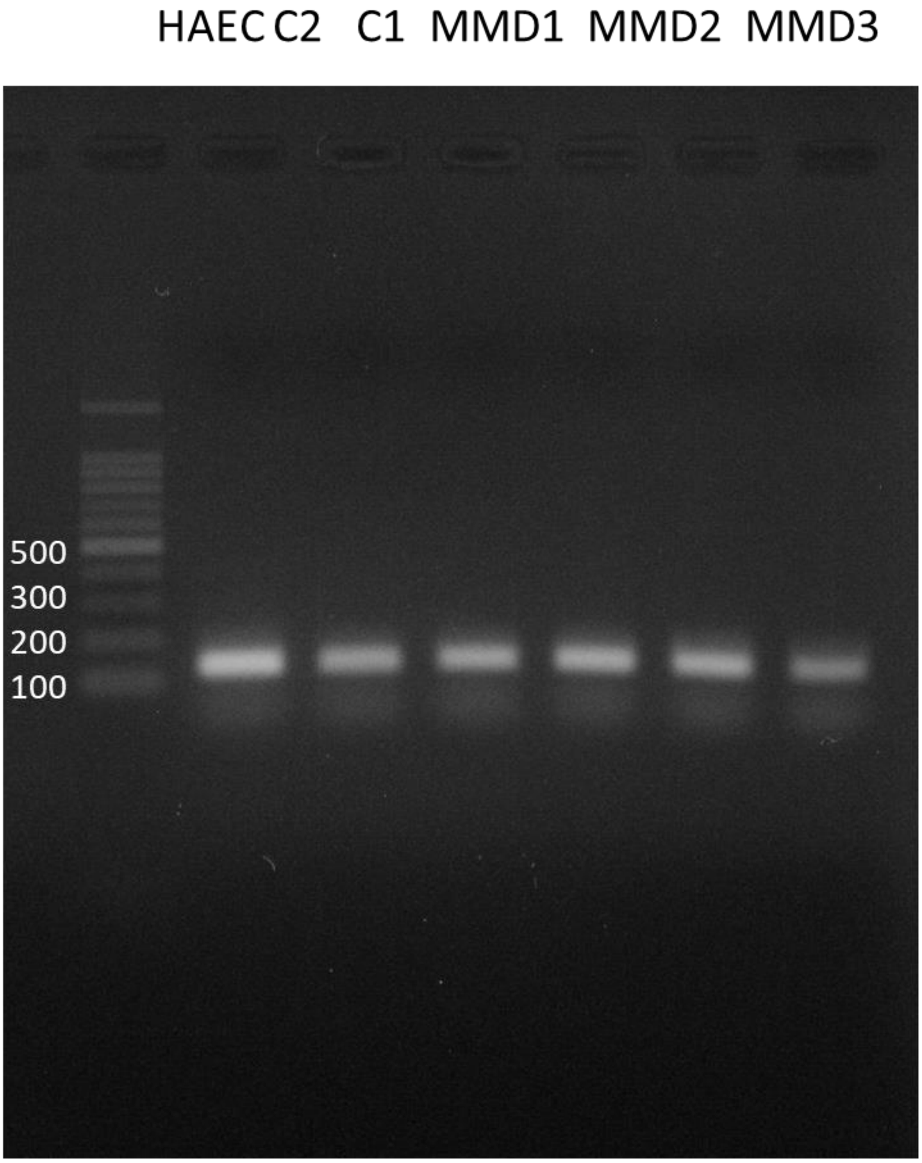

B. CD31

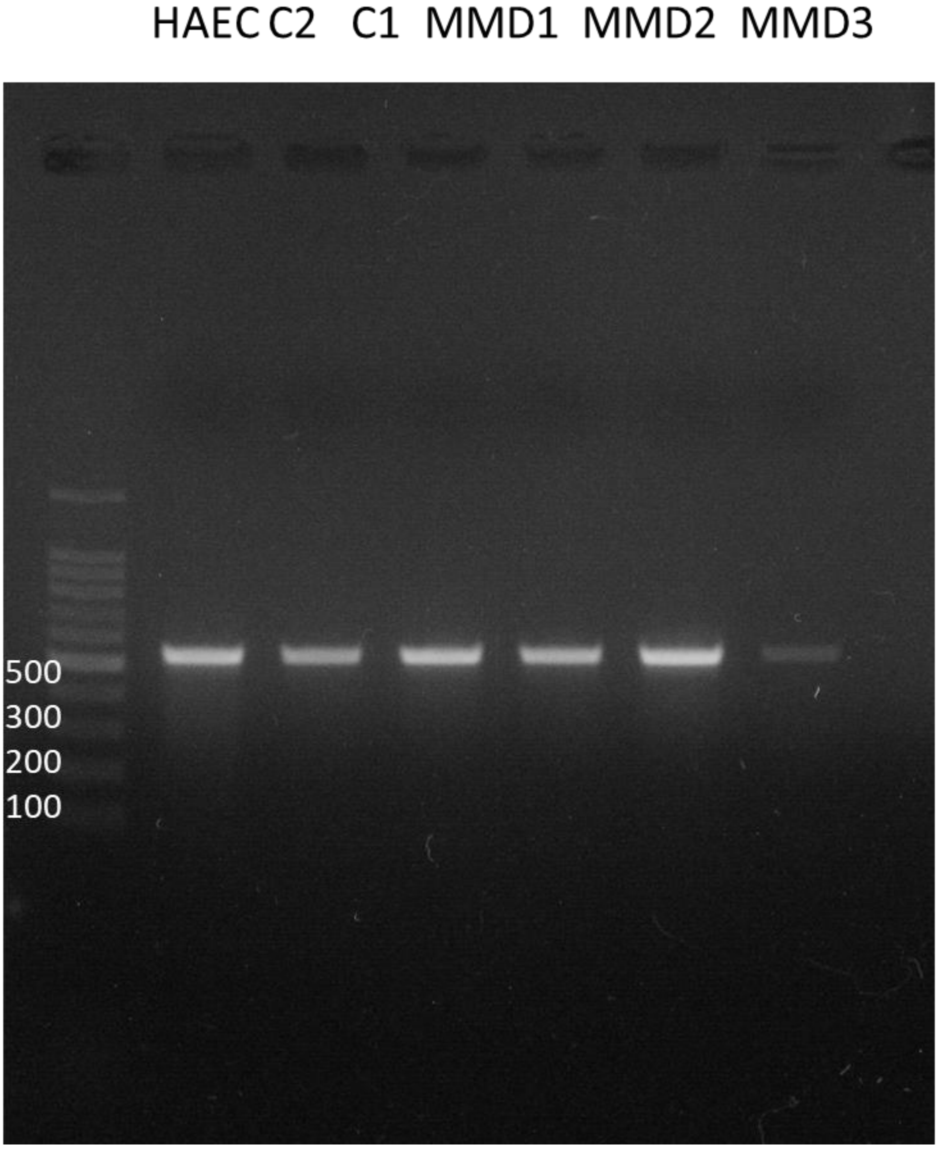

C. vWF

HAEC C2 C1 MMD1 MMD2 MMD3

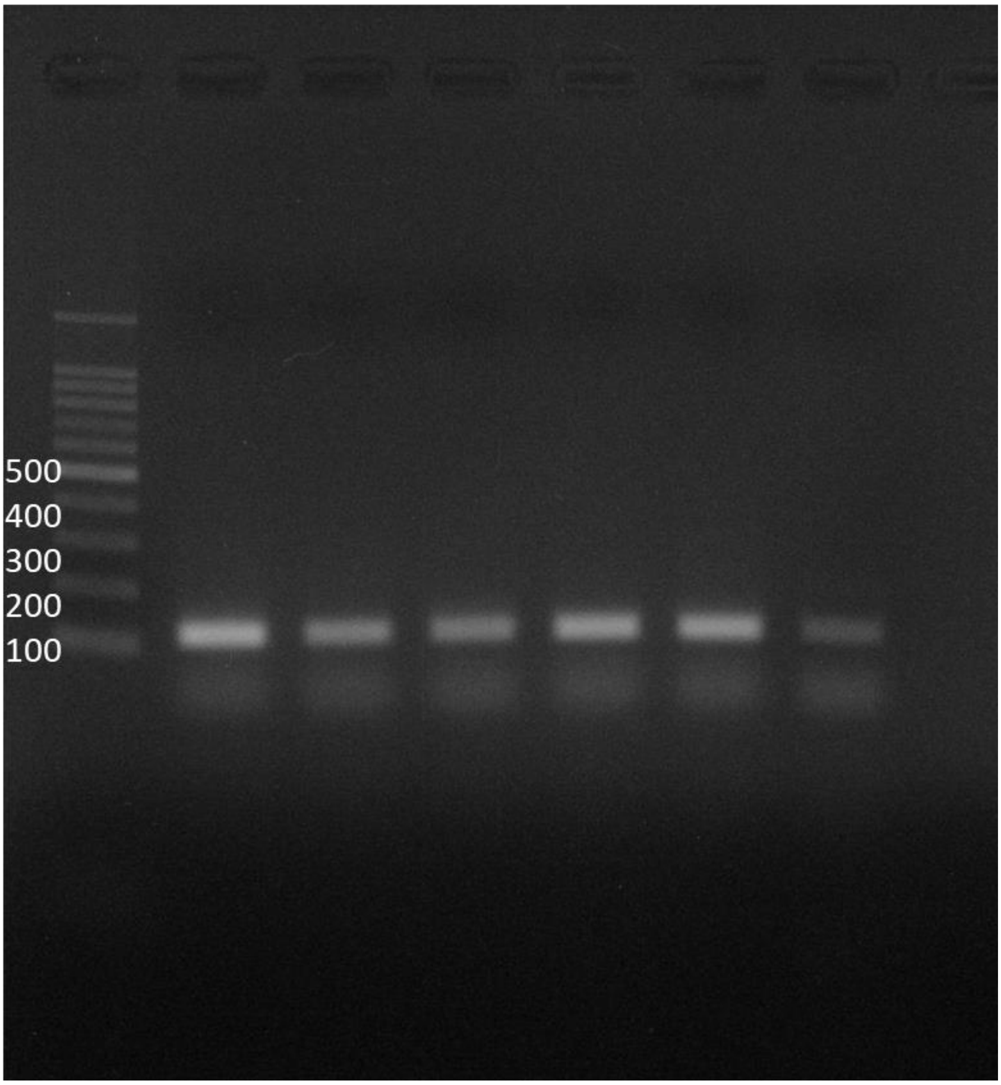

D. α-SMA

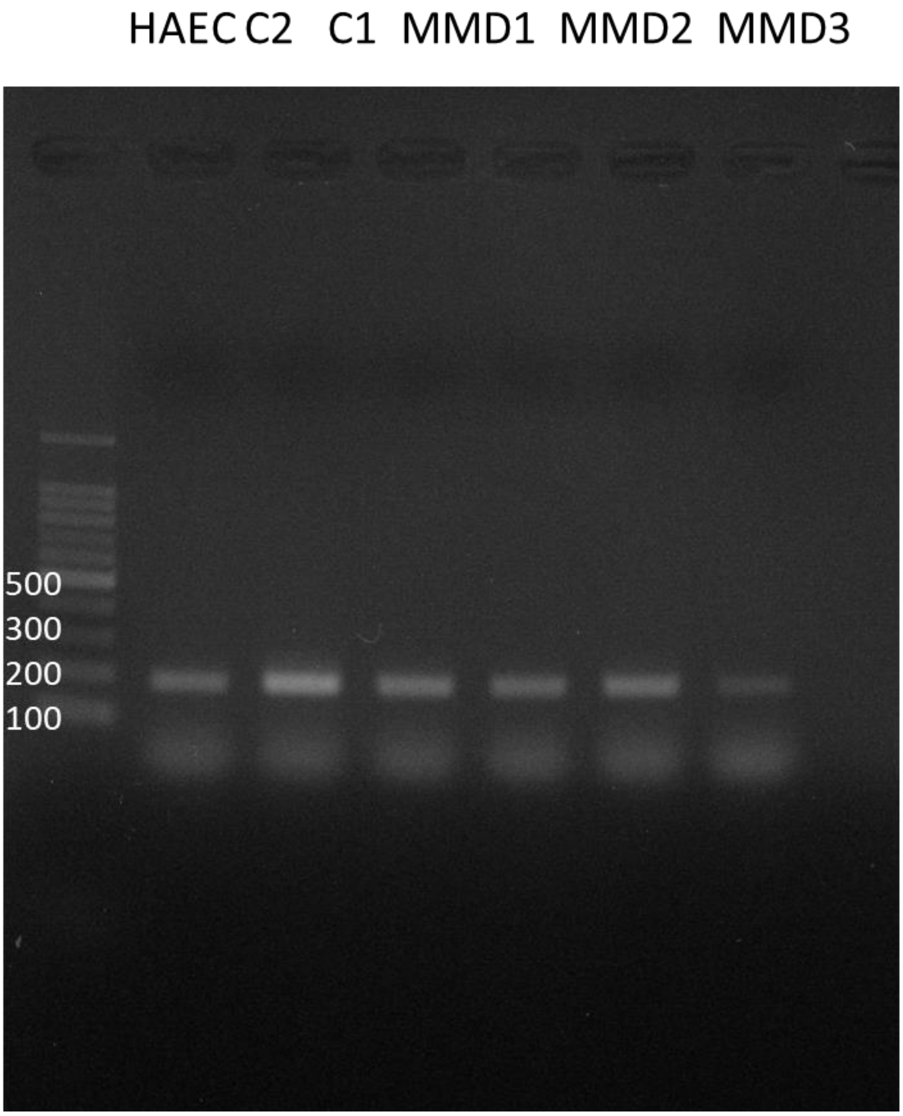

E. HAS2

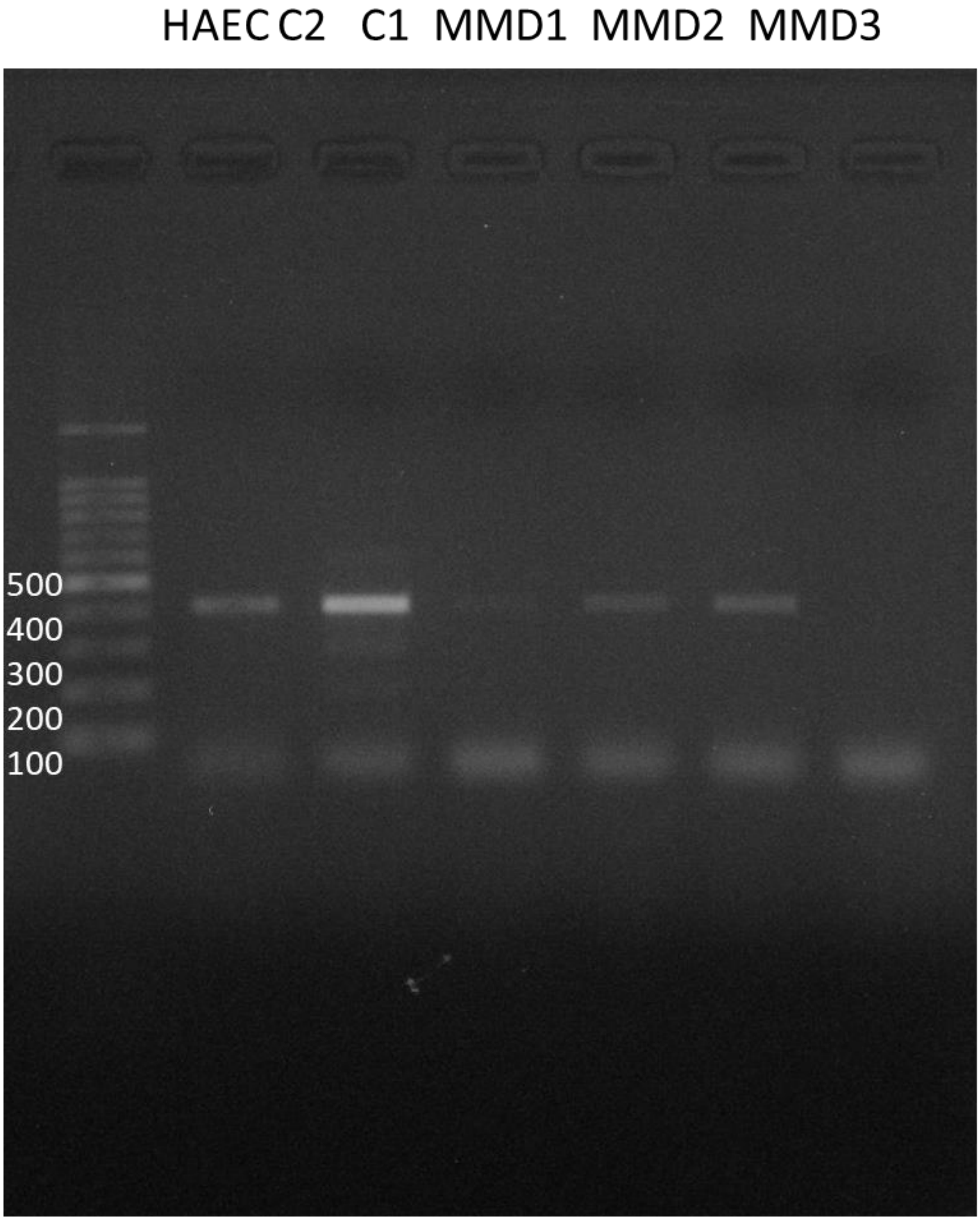

F. CD105

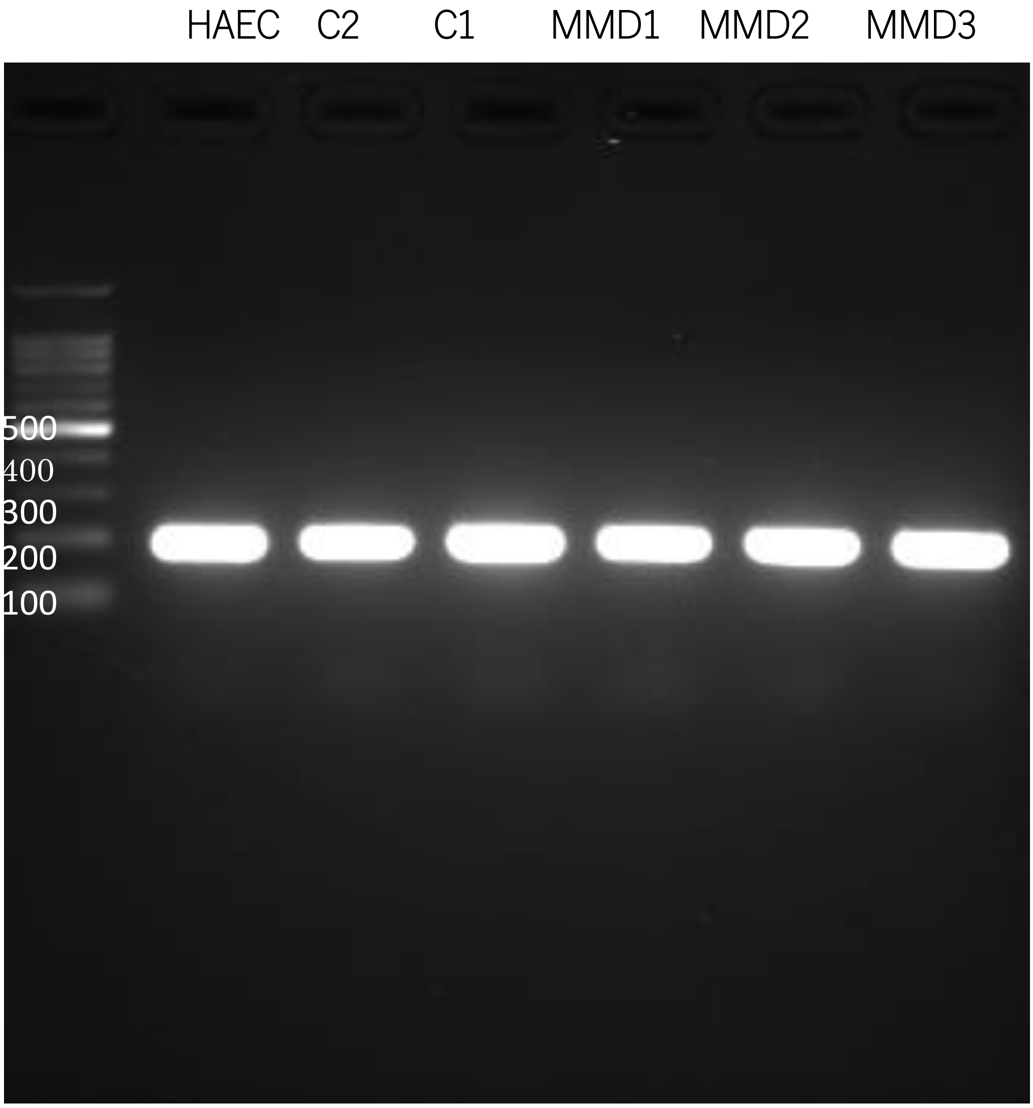

G. β–actin

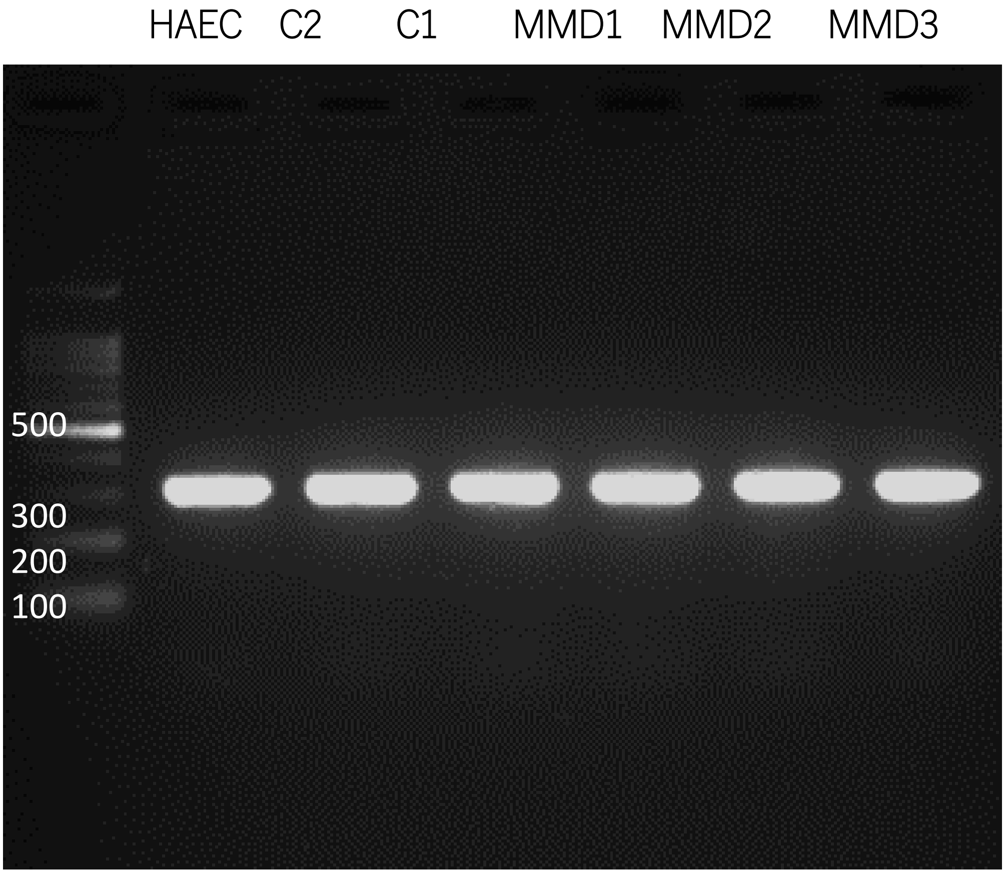

Figure S5. Network analysis by GeneMANIA

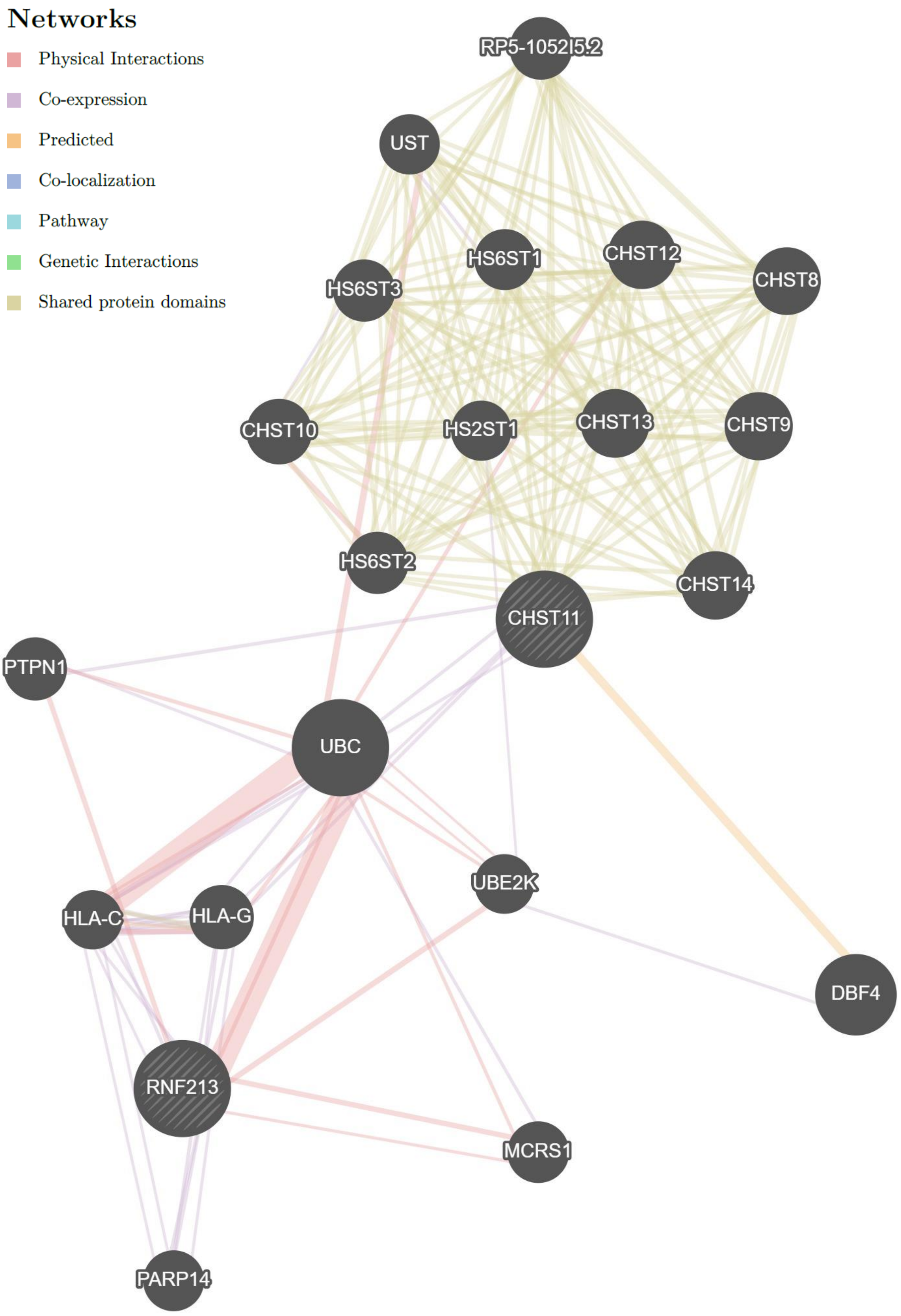

Gene	Description	Rank
RNF213	ring finger protein 213 [Source:HGNC Symbol;Acc:HGNC:14539]	N/A
CHST11	carbohydrate sulfotransferase 11 [Source:HGNC Symbol;Acc:HGNC: 17422]	N/A
UBC	ubiquitin C [Source:HGNC Symbol;Acc:HGNC:12468]	1
DBF4	DBF4 zinc finger [Source:HGNC Symbol;Acc:HGNC:17364]	2
CHST13	carbohydrate sulfotransferase 13 [Source:HGNC Symbol;Acc:HGNC: 21755]	3
CHST12	carbohydrate sulfotransferase 12 [Source:HGNC Symbol;Acc:HGNC: 17423]	4
CHST14	carbohydrate sulfotransferase 14 [Source:HGNC Symbol;Acc:HGNC: 24464]	5
CHST9	carbohydrate sulfotransferase 9 [Source:HGNC Symbol;Acc:HGNC: 19898]	6
CHST8	carbohydrate sulfotransferase 8 [Source:HGNC Symbol;Acc:HGNC: 15993]	7
CHST10	carbohydrate sulfotransferase 10 [Source:HGNC Symbol;Acc:HGNC: 19650]	8
HLA-G	major histocompatibility complex, class I, G [Source:HGNC Symbol;Acc: HGNC:4964]	9
PTPN1	protein tyrosine phosphatase, non-receptor type 1 [Source:HGNC Symbol;Acc:HGNC:9642]	10
RP5-1052I5. 2		11
HS6ST2	heparan sulfate 6-O-sulfotransferase 2 [Source:HGNC Symbol;Acc: HGNC:19133]	12
HS6ST3	heparan sulfate 6-O-sulfotransferase 3 [Source:HGNC Symbol;Acc: HGNC:19134]	13
MCRS1	microspherule protein 1 [Source:HGNC Symbol;Acc:HGNC:6960]	14
HS6ST1	heparan sulfate 6-O-sulfotransferase 1 [Source:HGNC Symbol;Acc: HGNC:5201]	15
PARP14	poly(ADP-ribose) polymerase family member 14 [Source:HGNC Symbol; Acc:HGNC:29232]	16
UST	uronyl 2-sulfotransferase [Source:HGNC Symbol;Acc:HGNC:17223]	17
HS2ST1	heparan sulfate 2-O-sulfotransferase 1 [Source:HGNC Symbol;Acc: HGNC:5193]	18
UBE2K	ubiquitin conjugating enzyme E2 K [Source:HGNC Symbol;Acc:HGNC: 4914]	19
HLA-C	major histocompatibility complex, class I, C [Source:HGNC Symbol;Acc: HGNC:4933]	20
